# Identification of Multiple QTLs Linked to Neuropathology in the Engrailed-1 Heterozygous Mouse Model of Parkinson’s Disease

**DOI:** 10.1038/srep31701

**Published:** 2016-08-23

**Authors:** Zuzanna Kurowska, Michael Jewett, Per Ludvik Brattås, Itzia Jimenez-Ferrer, Xuyian Kenéz, Tomas Björklund, Ulrika Nordström, Patrik Brundin, Maria Swanberg

**Affiliations:** 1 Neurodegeneration and Inflammation Genetics Unit, Wallenberg Neuroscience Center, Department of Experimental Medical Science, Lund University, BMC A10, Sölvegatan 17, 221 84 Lund, Sweden; 2 Department of Neurosciences, Lerner Research Institute, Cleveland Clinic, 9500 Euclid Avenue, Cleveland, Ohio 44195, USA; 3 Renovo Neural Inc., 10000 Cedar Avenue, Cleveland 44106, OH, USA; 4 Molecular Neuromodulation Unit, Lund University, Biomedical Center A10, Sölvegatan 17, 221 84 Lund, Sweden; 5 Department of Pharmacology and Clinical Neuroscience, Byggnad 6M NUS, Umeå University, 901 87 Umeå, Sweden; 6 Laboratory of Translational Parkinson’s Disease Research, Center for Neurodegenerative Science, Van Andel Research Institute, 333 Bostwick Ave, N.E., Grand Rapids, MI 49503, USA

## Abstract

Motor symptoms in Parkinson’s disease are attributed to degeneration of midbrain dopaminergic neurons (DNs). Heterozygosity for Engrailed-1 (En1), one of the key factors for programming and maintenance of DNs, results in a parkinsonian phenotype featuring progressive degeneration of DNs in substantia nigra pars compacta (SNpc), decreased striatal dopamine levels and swellings of nigro-striatal axons in the SwissOF1-En1+/− mouse strain. In contrast, C57Bl/6-En1+/− mice do not display this neurodegenerative phenotype, suggesting that susceptibility to En1 heterozygosity is genetically regulated. Our goal was to identify quantitative trait loci (QTLs) that regulate the susceptibility to PD-like neurodegenerative changes in response to loss of one En1 allele. We intercrossed SwissOF1-En1+/− and C57Bl/6 mice to obtain F2 mice with mixed genomes and analyzed number of DNs in SNpc and striatal axonal swellings in 120 F2-En1+/− 17 week-old male mice. Linkage analyses revealed 8 QTLs linked to number of DNs (p = 2.4e-09, variance explained = 74%), 7 QTLs linked to load of axonal swellings (p = 1.7e-12, variance explained = 80%) and 8 QTLs linked to size of axonal swellings (p = 7.0e-11, variance explained = 74%). These loci should be of prime interest for studies of susceptibility to Parkinson’s disease-like damage in rodent disease models and considered in clinical association studies in PD.

Parkinson’s disease (PD) is the second most common neurodegenerative disorder, occurring in more than 1% of people older than 65. Currently, clinical PD is based primarily on motor traits; nonetheless, PD patients also suffer from numerous non-motor symptoms that are a source of very significant morbidity^1^. The neuropathology of PD includes intraneuronal aggregates of alpha-synuclein (α-Syn) which are found in many brain regions^2^, but the motor symptoms are mainly attributed to reduced levels of dopamine in the striatum caused by degeneration of dopaminergic neurons (DNs) in substantia nigra pars compacta (SNpc)^3^,^4^. Although the number of approaches to alleviate PD motor symptoms is greater than ever before, none of the available therapies can slow disease progression.

Genetic analyses have identified several gene mutations in familial forms of PD, such as missense mutations in α-Syn and leucine rich repeat kinase 2 (LRRK2)^5^. However, in the large majority of patients, PD is idiopathic, i.e. caused by complex genetics in a multifaceted interplay between environmental and genetic risk factors^6^. So far, the most comprehensive genome-wide association meta-analysis on genetic risk factors for idiopathic PD has reported 24 potential risk loci^7^. Although each locus confers a small to moderate risk increment, the combination of risk alleles^7^ and interactions between specific alleles and environmental factors^8^ may significantly affect an individual’s risk for developing PD. The familial and the idiopathic forms most often share pathological features, e.g. misfolding and accumulation of α-Syn into Lewy bodies and degeneration of DNs in SNpc^9^. The underlying pathological processes are not fully understood, but there are lines of evidence in support of mitochondrial dysfunction^10^, neuroinflammation^11^, impaired axonal transport^12^, accumulation of reactive oxygen and nitrogen species^13^, as well as perturbed proteostasis due to changes in the lysosome-autophagy pathway or disrupted clearance of mis-folded proteins by autophagolysosomal pathways or the ubiquitin-proteasome system^14^,^15^. Therefore, efforts to identify targets for new, disease-modifying, PD therapy should aim at both the mechanisms behind degeneration of DNs and the integrity and function of striatal dopaminergic projections.

The development and subsequent maintenance of DNs require fine-tuning of a large number of genes. One of the annotated factors is Engrailed-1 (En1), a developmental gene of the homeobox family, essential for the programming of mesodiencephalic DNs in combinatorial action with pentraxin-related gene 3 (Pitx3) and Engrailed-2 (En2)^16^. En1 is expressed in all mesodiencephalic DNs from the one-somite stage and the expression persists until adulthood, controlling cell survival and maintenance in a cell-autonomous and dose-dependent manner^17^,^18^. Recent studies in mice have identified En1 and En2 as survival and regulatory factors for adult DNs^19^, and En2 has been shown able to compensate for loss of En1 activity, partly restoring the function and integrity of DNs^19^,^20^,^21^,^22^. Moreover, there are reports on genetic association between En1 polymorphisms and PD susceptibility^23^,^24^.

Studies in knock-out mice have revealed that the consequences of partial and complete En1 deficiency depend on the mouse strain, i.e. genetic factors outside the En1 locus^17^,^20^,^25^. While En1−/− bred on 129/Sv, C57Bl/6J×129/Sv or Swiss background is perinatally lethal with loss of colliculus and cerebellum, C57Bl/6-En1−/− mice are born alive^26^,^27^. Similarly, while SwissOF1-En1+/− mice display many parkinsonian characteristics, C57Bl/6-En1+/− mice appear normal and require addition of complete En2 deletion for developing degeneration of DNs^17^. The value of SwissOF1-En1+/− mice as a model for PD-like degeneration is strengthened with evidence of DN degeneration predominantly in the SNpc, and to a lesser extent in the VTA, loss of terminal synaptic function of DNs with reduced dopamine in the striatum, changes in autophagic function and presence of enlarged ovoid-like varicosities (here called ‘axonal swellings’) on dopaminergic nigrostriatal axons^20^,^28^. En1 is one of several developmental transcription factors that are maintained, and required for DN survival and/or axonal maintenance, in adulthood. Striatal DA signaling, autophagy and axonal integrity were recently found to be impaired also in Lmx1a/b genetic PD models^29^. This further strengthens the relevance of En1 mice as a model to study PD degenerative processes.

Linkage analysis is an essential tool to identify mutations in familial diseases and also to experimentally map complex traits in crosses of animal strains. In the case of neurodegenerative models, quantitative trait loci (QTLs) have been identified for neurodegeneration induced by nerve injury^30^,^31^, ethanol^32^ and excitotoxicity^33^. In PD models, susceptibility to MPTP- and paraquat-induced degeneration of DNs in SNpc has been mapped to three distinct QTLs in N2 back-crosses between toxin-sensitive C57BL/6J and toxin-resistant Swiss-Webster mice^34^,^35^. However, genetic mapping of susceptibility to spontaneous, progressive degeneration of DNs similar to the situation in PD patients has not been yet reported. The goal of this study was to map dopaminergic cell susceptibility loci by linkage analysis in the En1 heterozygous mouse model of PD.

Based on the strain differences in response to En1 deficiency, we hypothesize that the C57Bl/6 genome contains neuroprotective genetic variants affecting both axonal integrity and survival of DNs, and presents an opportunity to map genetic loci involved in these key pathogenic features of PD. We performed linkage analysis in En1+/− mice from a C57Bl/6^*^SwissOF1-En1+/− F2 intercross in order to map susceptibility alleles for DN degeneration and impaired nigrostriatal projections, both phenotypes of prime relevance to PD.

## Results

### Heterozygous disruption of En1 induces loss of dopaminergic neurons in Swiss-OF1 but not C57Bl/6

**Figure 1:**
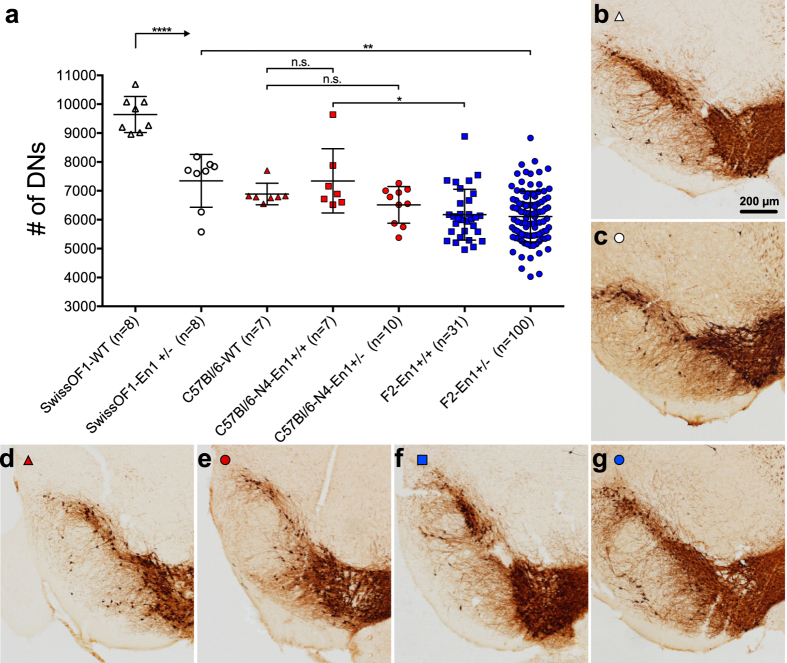
Neurodegeneration of TH-positive neurons in SNpc of the different mouse strains and intercrosses. (**a**) Stereological quantification of DNs in SNpc at 17 weeks of age. Individual data points and the mean +/− S.D. are shown. The mean number of DNs in SwissOF1-wt was significantly higher than in all other groups; ****p < 0.0001, **p < 0.01, *p < 0.05. C57Bl/6-N4 mice have in average 97% C57Bl/6 background genome. Representative images used for stereological quantification with TH-positive staining in SNpc of SwissOF1 wt(**b**), SwissOF1-En1+/− (**c**), C57Bl/6 wt (**d**), C57Bl/6-N4-En1+/− (**e**), F2-En1+/+ (**f**), F2-En1+/− (**g**). Scale bar: 200 μm.

Previous studies indicate that En1 heterozygosity leads to loss of nigral DNs in SwissOF1^20^, but not in C57Bl/6 mice^17^. We confirm a 24% loss of DNs in 17 week-old SwissOF1-En1+/− mice compared to wild-type (wt) mean number of cells 7346 and 9642, respectively, p < 0.0001 (Fig. 1a–c).

To address the effect of the same knock-out model on the C57Bl/6 background, we back-crossed SwissOF1-En1+/− males to C57Bl/6 females. Marker-assisted selection was used according to a speed-congenic approach, and mice from the fourth C57Bl/6 back-cross (N4) had an average of 3% SwissOF1 alleles outside the En1 locus. En1 heterozygosity in N4 mice did not induce degeneration of DNs in the SNpc: mean 6512 in N4-En1+/− and 7343 in N4-En1+/+ (Fig. 1a, e). The mean number of DNs in C57Bl/6 wt (6890) and N4 mice was in accordance with previous reports for C57Bl/6^36^, but as much as 29% lower than in SwissOF1 wt mice (9642, p < 0.0001, Fig. 1a, b, d). These data thus confirm loss of nigral DNs in En1+/− SwissOF1 mice, and show that the C57Bl/6 strain background confers protection to loss of nigral DNs after heterozygous deletion of En1.

### QTLs linked to loss of dopaminergic neurons in SNpc

**Figure 2:**
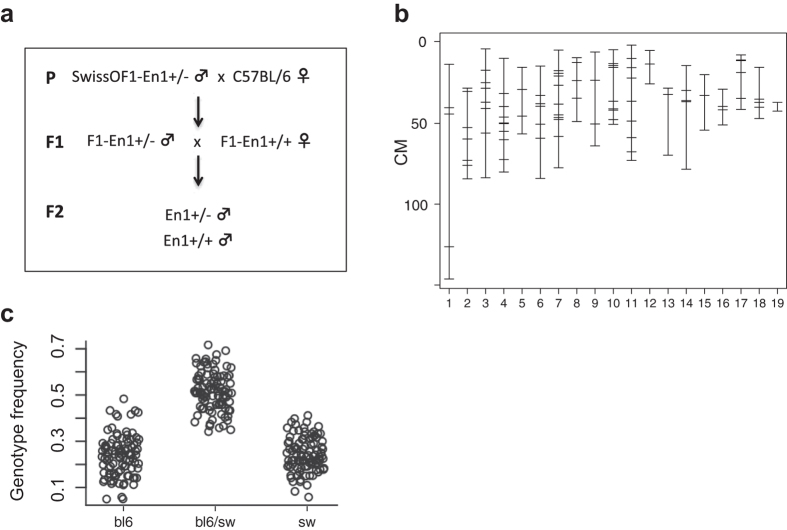
Genetic characteristics. (**a**) Parental (P) SwissOF1-En1+/− females and C57Bl/6 males were intercrossed to obtain the F1 generation, from which En1+/− females and En1+/+ males were crossed to obtain F2-En1+/− and F2-En1+/+ male mice; (**b**) Genetic map of F2 population showing physical location of informative SNP markers; (**c**) Genotype frequency by F2 individual.

SwissOF1-En1+/− males were intercrossed with C57Bl/6 females to generate F1 and F2 populations with En1+/+ and En1+/− genotypes (Fig. 2a). The F2-En1+/+ and F2-En1+/− populations had a similar mean number of DNs (Fig. 1a, f, g). The variance in the F2-En1+/+ group represents the segregation of alleles regulating the number of DNs in the respective wt parental strains, while the variance in the F2-En1+/− group also represents QTLs involved in the response to En1 heterozygosity. The mean number of DNs in F2-En1+/+ mice is similar to C57Bl/6 wt, but significantly lower than SwissOF1 wt. Similarly, the F2-En1+/− mean number of DNs is close to that of C57Bl/6-N4-En1+/−, but significantly lower than that of SwissOF1-En1+/− animals (Fig. 1a).

To identify loci linked to DNs susceptibility to degeneration in the absence of one En1 allele, the number of DNs in SNpc of F2-En1+/− mice at 17 weeks was used in genome-wide linkage analysis employing R-QTL^37^. Out of 377 genotyped SNPs, in the Illumina Mouse LD Linkage Panel, 114 were informative (Fig. 2b).

**Figure 3:**
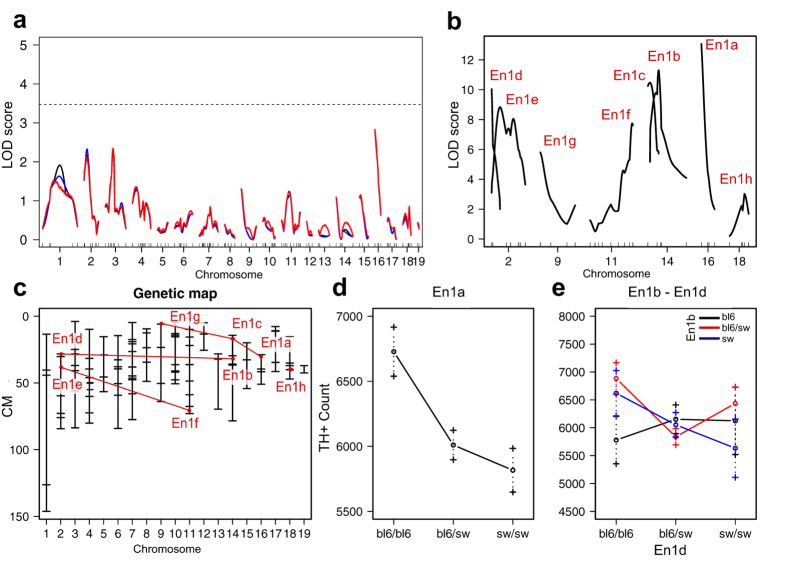
Results of the QTL analyses for the number of DNs in SNpc. (**a)** Single QTL scan reveals no QTLs. Solid black - expectation-maximization; red - multiple imputation; blue - Haley-Knott; dotted line: p = 0.05. (**b**) Multiple QTL scan reveals 8 significant QTLs named En1a-h. (**c**) Refined chromosomal locations of En1a-h from multiple QTL scan; red lines between interacting loci. (**d**) Effect-plot for En1a. (**e**) Interaction plot for En1b:En1d.

The phenotypic spread (Fig. 1a) suggested a complex genetic regulation of the trait. Single QTL analysis, which assumes a single QTL for the phenotype, revealed no significant peaks across the genome (Fig. 3a). We proceeded with a multiple QTL model and identified eight QTLs (En1a-h) linked to the number of nigral DNs (Fig. 3b, Table 1). The full multiple QTL model included interactions between seven of the loci (Fig. 3c) and explained 74% of the phenotypic variance (logarithm of odds (LOD) 28, p = 2.4E-9). Mice carrying two C57Bl/6 alleles in the most significant QTL, En1a, had an average of 6727 DNs compared to 6010 in heterozygous and 5816 in SwissOF1 homozygous mice (Fig. 3d). En1d showed a similar pattern, while En1g showed the opposite genotype effect (Suppl. Fig. 3). Notably, none of the identified QTLs showed any significant effect in the F2-En1+/+ cohort in QTL models or in single-marker analyses.

Pairwise interaction analyses are performed on groups defined by the genotype at two loci, with nine combinations for each pair. The most significant interaction pair was En1b:En1d (F = 5.8E-06). Among all genotype combinations, F2-En1+/− mice carrying C57Bl/6 alleles at both En1b and En1d display the lowest number of remaining DNs in SNpc, while those homozygous for C57Bl/6 alleles at En1d but heterozygous or SwissOF1 homozygous at En1b display the highest number of DNs (Fig. 3e). In the En1a:En1c interaction, there is a higher number of DNs in animals homozygous for C57Bl/6 only in combination with SwissOF1 alleles at En1c (Suppl. Fig. 3h).

Thus, no single QTL was linked to the number of DNs after heterozygous En1 disruption. Instead, SwissOF1 and C57Bl/6 alleles in eight distinct and interacting QTLs regulate the phenotype. In addition, these QTLs are specific to the susceptibility to En1 disruption and not related to differences in DN numbers between the wt strains.

### The effect of En1 heterozygozity on axonal defects in dopaminergic neurons

**Figure 4:**
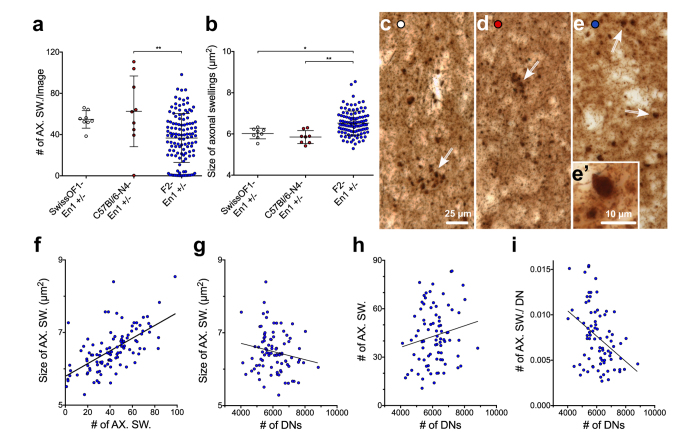
Quantification and size of axonal swellings. (**a)** Number of axonal swellings in parental SwissOF1-En1+/−, C57Bl/6-N4-En1+/−, F2-En1+/−. (**b**) Size of axonal swellings in parental SwissOF1-En1+/−, C57Bl/6-N4-En1+/−, F2-En1+/−. Axonal swellings indicated by arrows in dorso-lateral striatum of parental SwissOF1-En1+/− (**c**), C57Bl/6-N4-En1+/− (**d**) and F2-En1+/− (**e**). (**f**) Correlation of average size and number of axonal swelling (r = 0.62, p < 0.0001). (**g**) Correlation of average axonal swelling size vs number of DNs (n.s.). (**h**) Correlation of axonal swelling number vs number of DNs (n.s.). (**i**) Correlation of number of swellings per DN vs number of remaining DNs (r = −0.37, p < 0.001). **p < 0.01, *p < 0.05.

Axonal swellings were seen in the dorso-lateral portion of striatum in all 17-week old SwissOF1-En1+/− mice (Fig. 4a, c), but not in SwissOF1-wt or C57Bl/6-wt mice (data not shown). Swellings were also observed in most N4-En1+/− (7/8) and F2-En1+/− (105/120) mice. F2-En1+/− mice had, when comparing mean values, fewer but larger swellings compared to SwissOF1-En1+/− and N4-En1+/− mice (Fig. 4a–e).

### Correlation between axonal swellings and loss of DNs

In F2-En1+/− mice displaying axonal swellings, there was a positive correlation between the number of axonal swellings and the average size of the swellings (r = 0.62, p < 0.0001, Fig. 4f). Axonal swelling size was not correlated with the number of remaining DNs in the SNpc (Fig. 4g). We therefore consider axonal swelling size a possibly distinct phenotype for neuropathology in the En1 heterozygous mouse model.

In F2-En1+/− mice, the number of axonal swellings did not correlate to DN number (Fig. 4h). This can be explained by the fact that axons with swellings have been lost along with the respective soma of degenerated DNs, giving a low number of remaining axonal swellings in mice with severe neurodegeneration as well as in mice with little DN pathology. However, the more remaining DNs in the SNpc by 17 weeks of age, the less axonal swellings per neuron were seen (r = −0.37, p < 0.001, Fig. 4i). Thus, at the single timepoint studied here, axonal swellings are correlated with the number of DNs when taking previous loss of DNs into account.

### QTLs linked to the load of axonal swellings in the striatum

**Figure 5:**
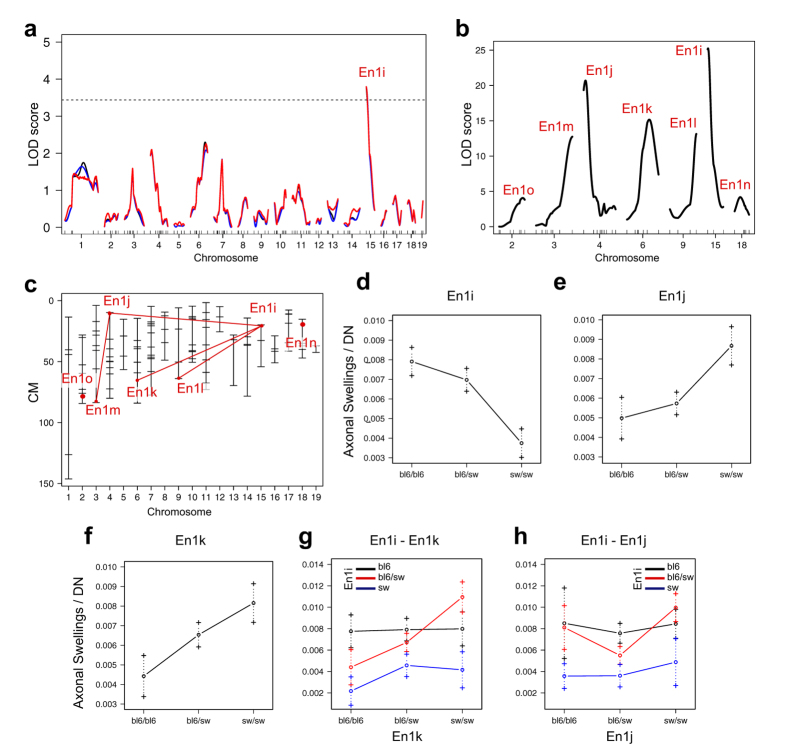
QTL analyses for the number of axonal swellings per number of remaing DNs. (**a**) Single QTL analysis reveals one QTL at chromosome 15. Solid black - expectation-maximization; red - multiple imputation; blue - Haley-Knott; Dotted line - p = 0.05; (**b**) Multiple QTL analysis reveals 7 QTLs; (**c**) Refined positions of the 7 QTLs from multiple QTL scan. Lines between interacting loci; (**d**–**f**) Effect of En1i (**d**) En1j (**e**) and Enk (**f**) on axonal swellings per number of remaining DNs; (**g**) Interaction plot for En1i and En1k; (**h**) Interaction plot for En1i and En1j.

Since axonal swellings appear in nigrostriatal neurons of En1+/− mice prior to degeneration of the DN soma, we used load of axonal swellings, i.e. the relative number of axonal swellings divided by nigral DNs number estimates at 17 weeks, as phenotype for linkage analysis. Single QTL analysis for the load of axonal swellings per remaining nigral DNs yielded one significant peak, En1i, located on chromosome 15 (Fig. 5a, Table 1). Interestingly, mice homozygous for SwissOF1 alleles at En1i displayed the lowest load of axonal swellings. C57Bl/6 alleles at this locus are thus linked to the presence of more axonal swellings on remaining DNs (Fig. 5d).

The phenotypic spread in F2-En1+/− (Fig. 4a) indicated the presence of additional QTLs, and multiple QTL analysis confirmed En1i and identified another six QTLs (Fig. 5b). Among these, En1j and En1k displayed the strongest effect. Opposite to the effect seen at En1i, SwissOF1 alleles in En1j and En1k were linked to the presence of more axonal swellings on remaining DNs (Fig. 5e, f)

The full model includes interactions between five out of the seven loci, with En1i interacting with En1j, En1k and En1l (Fig. 5c, Table 1). Comparing mice homozygous for C57Bl/6 and SwissOF1 alleles at En1i, two C57Bl/6 alleles at En1i lead to more swellings per remaining TH+ neuron regardless of the genotype of En1j and En1k (Fig. 5g, h). However, the phenotype in mice heterozygous at En1i depends on En1j and En1k, where mice homozygous for SwissOF1 alleles at En1j and En1k display the most swellings (Fig. 5g, h). The full model including seven QTLs and five interactions explained 80% of the phenotypic variance in the load of axonal swellings per remaining DN (LOD =  32, p = 1.7E-12, Table 1).

The load of axonal swellings is thus linked to one QTL, En1i, in a single model, and to seven, partly interacting, QTLs in a multiple model that explains the vast majority of the phenotypic variance of the trait.

### QTLs linked to the size of axonal swellings in the striatum

**Figure 6:**
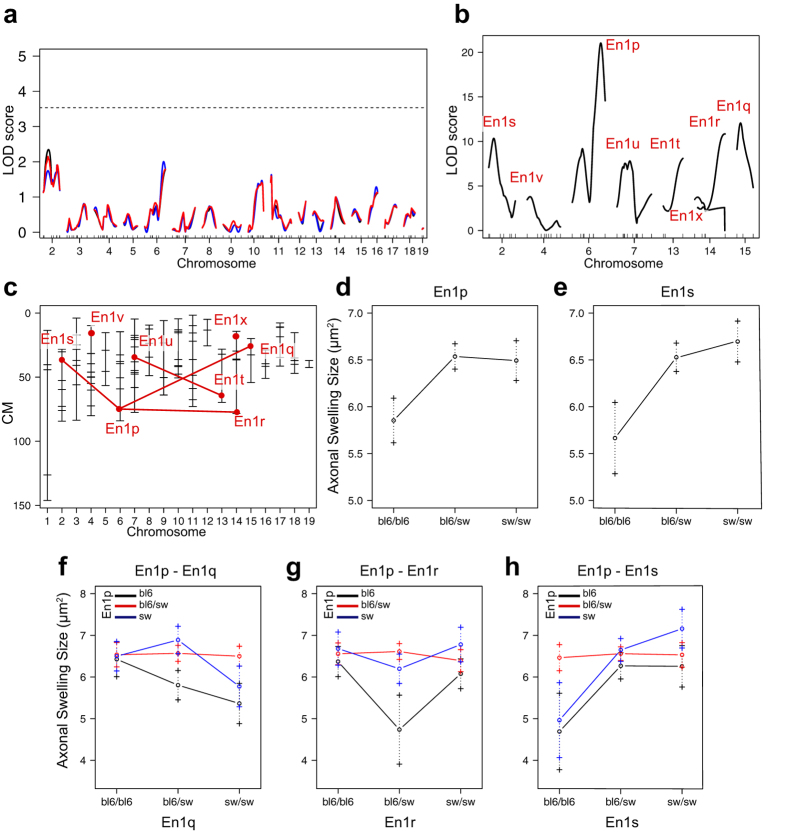
QTL analyses for the size of axonal swellings. (**a**) Single QTL analysis shown no hits. Solid black - expectation-maximization; red - multiple imputation; blue - Haley-Knott; Dotted line - p = 0.05; (**b**) Multiple QTL analysis revealed 8 different loci; (**c**) Refined positions of the 8 QTLs from multiple QTL scan. Lines between interacting loci; (**d**,**e**) Effect of En1p (**d**) and En1s (**e**) on size of axonal swellings; (**f**–**h**) Interaction plots for En1p and En1q (**f**), En1p and En1r (**g**) and En1p and En1s (**h**).

The group mean and variation in average size of axonal swellings was similar in SwissOF1-En1+/− and C57Bl/6, while both the average size and variation was greater in the F2-En1+/− population (Fig. 4b). Single QTL analysis did not identify any significant locus linked to the size of DN axonal swellings in the striata of F2-En1+/− mice (Fig. 6a), but the multiple QTL scan identified eight QTLs and interactions between six of these (Fig. 6b, c). The full multiple QTL model including interactions explained 74% of the phenotypic variance in the size of axonal swellings (LOD = 30, p = 7.0E-11, Table 1).

Effectplots show that homozygosity for C57Bl/6 alleles at En1p and En1s is linked to smaller swelling size (Fig. 6d, e). Moderate effects were seen from the other QTLs (Suppl. Fig. 5), likely due to the dependency of interacting QTLs as well as the relatively narrow range of the phenotype. En1p interacts with En1q, En1r and En1s, and C57Bl/6 homozygosity at En1p is linked to smaller swelling size in combination with SwissOF1 alleles at En1q (Fig. 6f), heterozygozity at En1r (Fig. 6g) and C57Bl/6 homozygozity at En1s (Fig. 6h).

### Overlapping QTLs

Overlapping QTLs for the two axonal swelling phenotypes were found on chromosome (chr) 4 (En1j and En1v), and QTLs with estimated positions in close proximity to each other were found on chr 6 (En1k and En1p) and chr15 (En1i and En1q). The direction of effect were similar in each pair, i.e. SwissOF1 alleles was linked to more swellings and larger swelling size for En1j-En1v and En1k-En1p, and fewer swellings and smaller size for En1i-En1q. Thus, the same QTL linked to both average size of and number of axonal swellings per remaining DN may underlie the respective two QTLs on chr 4, 6 and 15.

Other possibilities for shared QTLs are En1h and En1n on chr 18 (Suppl. Figs 3g and 4c), where heterozygozity in the locus resulted in higher number of DNs and smaller size of axonal swellings, and En1d, En1e and En1s on chr 2, where mice homozygous for C57Bl/6 alleles display higher DN counts and lower number of axonal swellings (Suppl. Fig. 3c, d and Fig. 6e).

The neuroprotective effect of En1c on chr 14 with heterozygous mice displaying the highest average of DNs was, however, not reflected in the nearby locus En1b, suggesting at least two separate effects of these loci (Suppl. Fig. 3).

## Discussion

Although susceptibility to neurodegeneration is a complex genetic trait known to be strain-dependent in mice, this study is the first to genetically map loci regulating PD-like neuropathology in a spontaneous PD mouse model. We identify QTLs that explain the vast majority of the variation in DN degeneration and axonal pathology by linkage analysis in an F2 intercross with disruption of one En1 allele (En1 hemizygous). The F2 intercross was obtained from SwissOF1 mice, that display PD-like pathology with preferential loss of DNs in SNpc when En1 hemizygous, and C57Bl/6 mice that do not display DN degeneration when En1 hemizygous. We chose to map three distinct features of PD-like pathology: loss of DNs in SNpc, load of swellings on DN axons, and size of swellings on DN axons. While analyses assuming the presence of a single QTL per phenotype only revealed a significant QTL for the load of axonal swellings (En1i), multiple QTL analyses revealed several loci with high LOD scores and interactions for all three phenotypes. A large number of QTLs and interactions between loci are typical for complex traits. Knowing that the etiology of 90% of PD cases is complex with multiple interacting genetic and environmental risk factors, the presented QTLs linked to PD-like pathology in the En1 mouse model are particularly relevant to idiopathic PD.

We replicated loss of about 20% of DNs in SNpc in four month-old SwissOF1 mice lacking one En1 allele^20^,^28^. The total number of DNs estimated in our SwissOF1-En1+/− cohort was similar to that reported by Sonnier *et al*. but around twice the number reported by Norsdström and colleagues. The reasons behind this discrepancy are likely related to parameters used in stereological cell counts, such as delineation of region of interest (Suppl. Fig. 1), use of guard zones, thickness of sections, TH detection methodology, or differences between colonies of these mice. Interestingly, the number of DNs in the SNpc of C57Bl/6 mice (C57Bl/6-wt and C57Bl/6-N4-En1+/+) was significantly lower compared to SwissOF1 wt. These differences in DN numbers between adult mice of different inbred strains could be attributed to a different rate of early developmental neurogenesis/apoptosis or age-related cell death in the mesencephalon in the two strains. Like Sgado *et al*., we saw no loss of DNs in En1-heterozygous N4 mice having a large majority of C57Bl/6 alleles in their genetic background^17^. Thus, despite a lower starting number of DNs, mice with C57Bl/6 genetic background were more resistant to En1 depletion.

We report a similar average and variation in number of DNs in F2 mice with and without partial loss of En1, and the numbers are lower than those in each parental group. Considering that En1 is part of a complex network of transcription factors that orchestrate the development and survival of DNs^16^, it is not surprising with a large variation in the number of DNs between genetically heterogenous F2 mice with only one En1 allele. Other important factors contributing to the phenotypic variation in the F2 population are differences in DN numbers between the wt parental strains SwissOF1 and C57Bl/6, and transgressive segregation, which may cause extreme phenotypes in hybrid populations^38^. The reported QTLs linked to DN number likely represent both innate strain differences and strain-specific responses to En1 hemizygosity, but single-marker analysis for QTLs identified in F2+/− did not show any significant effects in the relatively small F2-En1+/+ cohort.

The multiple QTL models described here all had LOD scores that far exceeded the significance thresholds given by permutation tests. Some QTLs in the models had LOD scores below the significance threshold for individual QTLs. However, this threshold was estimated from the distribution of the max LOD score, rather than all LOD scores, in each permutation. In addition to contributing to the strength of the full model, some of the QTLs below the threshold were overlapping with significant QTLs for other phenotypes. When QTLs for different phenotypes in a model overlap, they may represent shared or neighbouring alleles that regulate mechanisms of key importance to the model, in this case DN integrity and survival. We found indications of such shared QTLs on chr 2 (En1e, En1s), chr 4 (En1j, En1v), chr 6 (En1k and En1p), chr15 (En1i and En1q) and chr 18 (En1h and En1n). Thus, the number of QTLs reported here likely are over-estimated, and the loci with impact on both axonal pathology and DN survival may regulate key features of DN integrity and function and are highly interesting candidate regions for fine-mapping. A limitation to this study is the one-directional cross, preventing analyses of founder effects. Some of the identified QTLs may thus be specific to a SwissOF1-En1+/− male to C57Bl/6 female, and not a reciprocal cross.

En1 polymorphisms in humans have previously been suggested to be associated with PD risk^23^,^24^. These were relatively small studies and they would need to be replicated in larger cohorts to be conclusive. However, mouse models have demonstrated the relevance of En1 to PD. SwissOF1 mice with En1 disruption display, among other PD-like phenotypes, progressive degeneration of DNs^20^,^28^. In addition to the loss of midbrain DNs, these mice exhibit several neuropathological features that are analogous to those seen in the brains of PD patients. For example, the SNpc neurons are affected earlier and to a greater extent than the adjacent VTA DNs. The loss of En1 function is associated with mitochondrial deficits, akin to what is observed in PD. Moreover, multiple changes in the mTOR pathway, which controls e.g. autophagy, appear in En1 mice. Observations of ultrastructural changes in axonal swellings that are concurrent with accumulation of autophagic vacuoles in the nigrostriatal pathway^28^ and similar phenotypes described in Lmx1a/b deficient mice^29^ suggest that DNs undergo a dying back process with several features resembling what has been proposed to occur in PD^39^,^40^. Furthermore, before DNs degenerate in the En1+/− model, their capacity to release and take up dopamine is dramatically impaired in parts of the striatum^28^, similar to what is suggested to occur in PD. Moreover, there is evidence for marked neuroinflammation in the SNpc of En1+/− mice (Ghosh *et al*. in preparation), similarly to what is seen in PD. Finally, the En1 protein has recently been reported to protect against mitochondrial insult and oxidative stress in DNs, which is of particular interest considering that oxidative stress has been strongly implicated as a pathogenic mechanism in PD^41^. Aside from SwissOF1, the single allele knock-out of En1 has been shown to cause neurodegeneration in other inbred mouse strains^17^,^20^,^25^ but no degeneration in mice with C57Bl/6 background^17^. A possible compensatory mechanism could act through En2, which has been shown to be able to compensate for En1^19^,^20^,^21^,^22^. Based on our linkage analysis, however, we conclude that there is no cis-acting effect of C57Bl/6 alleles in the En2 locus, since none of the QTLs overlap the position of the En2 gene. There might, however, be trans-acting effects from distal QTLs that regulate En2 gene expression, transcript stability, translation, or protein activity. It should also be emphasized that possible QTLs in close proximity to the En1 gene on chromosome 1 could not be assessed in the F2 cohorts studied. This is due to transgene selection at the En1 locus, leading to alleles from the original transgene surrounding the En1 gene and a distorted genotype distribution (no C57Bl/6 homozygozity) in the transgenic region. Therefore, we cannot rule out the possibility that C57Bl/6 alleles close to the En1 knock-out transgene impacted the phenotypes studied here.

While the loss of DNs in SNpc is the classical hallmark of PD, recent research suggests that axonal changes in the nigrostriatal neurons precede cell loss. It has been proposed that Wallerian-like axonal degeneration is a common feature of various neurodegenerative disorders^42^,^43^. In a study on PD human brains, the levels of TH and dopamine transporter (DAT) in axons were vastly depleted in the putamen, and were virtually gone within the next 4 years of diagnosis (i.e. following onset of motor symptoms), whereas the loss of cells in the SNpc progressed most rapidly during the decade following PD recognition^40^. In addition, dystrophic axonal spheroids with accumulated beta- and gamma-synuclein have been found in the hippocampus of PD patients^44^. These post-mortem studies support the notion that axonal failure is a significant prodromal hallmark of PD, and these are also reflected in animal PD models^12^. En1 heterozygous mice with the SwissOF1 background display abnormal TH-positive axonal swellings as early as 8 days after birth. These increase in number and size over the following weeks, and exhibit accumulations of mitochondria and electron-dense vacuoles, suggesting dysfunction in axonal transport, autophagy or faulty synapse maintenance^28^. No axonal abnormalities were reported in previous studies on En1 heterozygous mice with C57Bl/6 genetic background^16^,^17^, even when mice were studied until old age^17^. In the present study, we did not observe cell loss in 17-week old C57Bl/6-N4-En1+/− mice, but nonetheless striatal axonal swellings were as abundant as in SwissOF1-En1+/− mice of the same age. This could be interpreted as a sign of delayed nigrostriatal degeneration in En1 heterozygous C57Bl/6 mice compared to SwissOF1, and it cannot be excluded that older N4-En1+/− mice would exhibit nigral cell death. In the F2-En1+/− population analyzed here, we see a large inter-individual variation in both the neurodegenerative phenotype and in the load of axonal swellings. By correlating these phenotypes, we conclude that the more DN somas remain in the SNpc, the fewer axonal swellings the DNs have, suggesting that surviving DNs could be protected from both soma and axon degeneration. Alternatively, the population of degenerating vs. surviving DNs may belong to different subtypes^45^. Linkage analysis did, however, identify both unique and overlapping QTLs for these two traits arguing for them being biologically related pathological processes. To better understand the nature, causes and consequences of axonal swellings appearance, further investigation involving e.g. neuroprotective agents is needed.

Although finer mapping is necessary to pinpoint the specific genes underlying the QTLs, there are a couple of candidates, in or in close proximity to the identified loci, which have previously been indicated in PD pathogenesis. Recently, structures almost identical to the axonal swellings in En1+/− mice: TH-stained “abnormally large profiles” and “enlarged presynaptic boutons,” were observed in the striata of adult Lmx1a/b conditionally-depleted mice, and they were accompanied by loss of dopamine in the striatum, loss of DNs in SNpc and VTA and other changes resembling both PD and the phenotype of SwissOF1-En1+/− mice^29^. Since Lmx1b lies close to En1d identified in this study, it is a strong candidate for harboring allelic differences affecting susceptibility to En1-heterozygosity. Another QTL on chromosome two, En1o for number of axonal swellings per DN is close to the Foxa2 gene. Mice carrying only one copy of the Foxa2 gene show abnormalities in motor behavior in old age and an associated progressive loss of dopamine on the Swiss background^46^. Otx2, located on chromosome 14, within En1x and in proximity of En1b and En1c, is another candidate that could be influential in our experimental paradigm. It is a homeobox transcription factor that is expressed in nigral DNs only during development. Mild over-expression of Otx2 in SNpc progenitors and neurons was sufficient to rescue En1 haploinsufficiency-dependent defects, such as progressive loss of SNpc neurons^47^. A hypothesis linking defective autophagy to axonal swellings is supported by the fact that the gene encoding Atg7, a necessary component of the autophagy process, is located close to En1k on chromosome 6. Studies have shown that conditional deletion of Atg7 in nigral neurons leads to age-dependent loss of DNs and corresponding loss of striatal dopamine^48^. En1k is linked to the number of axonal swellings per remaining neuron and F2-En1+/− carriers of C57Bl/6 alleles at this locus display almost half as many swellings per DN as homozygous Swiss-allele carriers (Fig. 5f). This is an indication of a more efficient autophagy process in the C57Bl/6 compared to Swiss strain, possibly underlying part of the protection to En1-induced pathology in DNs.

Due to the complex genetic structure of idiopathic PD, we emphasize the importance of understanding the genetic regulation of dysfunction and degeneration of DNs in order to better understand disease etiology and to develop new therapies. The present mapping of QTLs linked to DN loss and axonal pathology identifies multiple interacting QTLs linked to these phenotypes and offers the possibility for genetic fine-mapping. Potential candidate genes from our study are of prime interest to validate by QTL fine-mapping, functional studies in culture and *in vivo* and by gene targeting. These genes will provide new clues to biologically relevant mechanisms of PD, which is needed to identify new neuroprotective strategies to increase survival and function of DNs and alleviate patient’s symptoms.

## Methods

### Animals and breeding schemes

All procedures described were approved by the Ethical Committee for the use of laboratory animals in the Lund/Malmö region and were conducted in accordance with the relevant guidelines and regulations. A schematic drawing of the breeding strategy is shown in (Fig. 2a). The En1+/− strain was generated as described earlier^20^ and bred on the SwissOF1 background (Charles River). To generate an F2 population, C57BL/6NCrl (C57Bl/6) males (Charles River) were crossed with SwissOF1-En1+/−females. From the F1 generation, En1+/+ males were crossed with En1+/− females to produce the F2 generation. In total, 129 F2-En1+/− and 57 F2-En1+/+ males were sacrificed at 17 weeks of age. In order to study C57Bl/6 mice with a single allele knockout of En1, the disrupted En1 locus was transferred from SwissOF1-En1+/− to the C57Bl/6 background with a speed congenic approach consisting of repeated backcrossing to C57Bl/6 females (Charles River) with marker-assisted selection^49^. The backcross started with an F2-En1+/− male. In each generation, En1+/− male mice were subjected to single nucleotide polymorphism (SNP) analysis (Illumina Golden Gate assay^50^) to estimate the fraction of C57Bl/6 background in the genome. The En1+/− male with the highest number of C57Bl/6-alleles was kept for back-crossing with C57Bl/6 females to produce the next generation. The phenotyped C57Bl/6-N4 generation had an average of <3% SwissOF1 alleles outside the En1 locus.

### DNA isolation

Ear punches, tail tips or brain tissue were incubated in 0.5 mL lysis buffer (trizma base (1 M, pH 8.5), edetic acid (0.5 M), sodium dodecyl sulfate (10%), sodium chloride (5 M) and Milli-Q water (Millipore Corporation) and 2.5 μL Protease K (20 mg/ml) at 55 °C while shaking at 600 rpm for either 1 h for ear or 2 h for tail and brain biopsies. Once the tissue was lysed, it was centrifuged at 14,000 rpm at 4 °C for 10 min. The supernatant was transferred to 0.5 mL ice-cold isopropanol, and the DNA was precipitated by gently shaking the tubes. At this point, the samples were centrifuged again at 14000 rpm at 4 °C for 10 min, then all liquid was removed and tubes were put to dry in a ventilated hood at room temperature (RT) for at least 1 h. Once dry, 100 μL Milli-Q water was added, and the samples could then be incubated at 37 °C overnight to dissolve the pellet. The DNA was subsequently used for both genotyping of En1 and genome-wide SNP genotyping.

### Genotyping En1 knockout

To identify the single allele knockout of En1, we performed PCR with primers for LacZ, the gene used for deleting En1 allele. The PCR was performed by mixing 2 μL DreamTaq Green Buffer (ThermoScientific), 0.5 μL dNTP, 13.3 μL Milli-Q water, 1 μL LacZ Forward primer (5′-TGT ATG AAC GGT CTG GTC TTT G-3′, 10 μM), 1 μL LacZ Reverse primer (5′-AAC AGG TAT TCG CTG GTC ACT T-3′, 10 μM), 0.2 μL Taq polymerase (# EP0702, Thermo Scientific) and 2 μL genomic DNA (10 ng–1 μg). The PCR product was 128 bp long and was identified by gel-electrophoresis on a 2% Agarose gel.

We also applied the SsoAdvanced^tm^ SYBR^®^ Green Supermix (Bio-Rad) for genotyping of LacZ. By performing qPCR with melting curve assay at the end, we could assess the presence of a product. The qPCR was performed by mixing 10 μL of SsoAdvanced_tm_ SYBR® Green Supermix, 0.6 μL of LacZ forward and 0.6 μL LacZ reverse primers, 50 ng–5 pg DNA, and Milli-Q water for a total volume of 20 μL. After perfusion and brain harvesting (as described below), each cerebellum piece was incubated while shaking in dark conditions over-night at RT in 2 μL MgCl_2_, 25 μL X-gal (40 mg/μL), 10 μL K_3_Fe(CN)_6_ (0.5 M), 10 μL K_4_Fe(CN)_6_ (0.5 M) and 0.955 mL PBS-T (0.3%).

### Perfusion and brain dissection

At 17 weeks of age (+/− 3 days) mice were sedated by intraperitoneal injection of 0.2 mL sodium pentobarbital (40 mg/μL), before being perfused through the ascending aorta with ice-cold saline (0.9% NaCl) for 3 minutes. After isolating the brain, the cerebellum was sliced off and post-fixed in PFA (4%, pH 7.4) for 20 minutes and then transferred to saline for subsequent LacZ staining. The remaining brain was placed in a mouse brain slice matrix and cut sagitally down the midline with a fine razor blade. The left hemisphere was immediately placed in 10–15 mL PFA (4%, pH 7.4) and post-fixed overnight and subsequently cryoprotected in 30% sucrose (in PBS, with 0.01% sodium azide).

### Immunohistochemistry

The left hemispheres of the dissected brains were sectioned coronally on a freezing microtome (Leica SM2010R) at 40 μm. Immunohistochemical stainings were performed on free-floating sections. The SNpc sections, were given an initial antigen-retrieval incubation in Tris/EDTA (pH 9.0) at 80 °C for 45 min. All the sections were quenched with 3% H_2_O_2_/10% MetOH for 30 min then blocked with 5% Normal Horse Serum (NHS) for SNpc and Normal Goat Serum^29^ for Striatum before overnight RT incubation with primary antibody (mouse anti-TH 1:10000 for SNpc, Immunostar, Wisconsin for SNpc; rabbit anti-TH 1:4000, Millipore, California for Striatum). On the second day, sections were incubated with the corresponding biotinylated secondary antibody (horse anti-mouse 1:200; goat anti-mouse 1:200, Vector Laboratories) for 1 h at RT. This was followed by a 30-min incubation with an avidin-biotin peroxidase solution (ABC Elite, Vector Laboratories), and the antigen was visualized using 3,3-diaminobenzidine (DAB) as a chromogen. Sections were mounted on glass slides, dehydrated with increasing concentrations of ethanol and pure xylene, and finally coverslipped using DPX mounting medium (Sigma-Aldrich, Gillingham). Sections with uneven, blurry, not penetrating staining were excluded from analyses.

### Stereological estimates of nigral neurons

Stereology was performed according to the optical fractionator principle in order to quantify the total number of tyrosine hydroxylase-positive DNs in the SNpc. We used Leica microscope connected to digital camera (Leica MPS52) employing Stereo Investigator software (MBF Bioscience). Every third section (section sampling fraction, ssf = 3) of the midbrain region (Bregma −2.70 to −3.78 51) was analyzed (Suppl. Fig. 1a–i), which yielded between 9–11 sections per animal. Tracing regions of interest (ROIs) was done using the 5X/0.11 lens, and counting was performed with 100X/1.30 lens. The average mounted section thickness (h) was 23.6 μm (+/−2.6) and no guard zones were used due to variable in section thickness (thickness sampling fraction, tsf = 1). Section thickness was measured at every fourth site while counting and the area-sampling fraction (asf) was on average 0.0883. Dissector volume (h*A_frame_) was 60,500 mm^3^ on average, and the average number of DNs counted in each individual was 234 (+/−48). A maximal Gundersen coefficient of error (CE)^52^ of 0.08 was accepted, and the smoothness factor (m) of 1 was used. The following morphological criteria had to be fulfilled in order for the cell to be included: the cell body was highly defined with a visible nucleus due to fewer TH particles available; a darker stained cell may have not meet this criteria, but its projections were distinctly visible making it clear that the stained particle is a cell (Suppl. Fig. 1j). 29 of 129 F2-En1+/− and 26 of 57 F2-En1+/+ were excluded from the analysis due to complications with tissue processing, leaving 100 F2-En1+/−and 31 F2-En1+/+ for quantification. A genotype-blind operator performed the stereological assessment. To test for normal distribution of cell numbers among F2 generation, the Shapiro-Wilk normality test in R (3.0.2) was used.

### Axonal swelling quantification

Three to five consecutive sections from each animal at bregma distance 0.72–0.92 mm were stained and analyzed. High-resolution 25x pictures were taken using the same microscope, camera and software as for the stereology. Four pictures were taken of precisely delineated ROIs spanning an area of about 3.6 mm^2^ representing the dorso-lateral part of the caudate putamen of the striatum, the main functional regions of the nigrostriatal pathway^28^, for every section (Suppl. Fig. 1k). Image J was used to identify the swellings and calculate their total number, as well as their size, by setting an exclusion threshold for particles <3 μm^2^. The average number and size of swellings were calculated based on all pictures from one animal. Nine of F2-En1+/− samples were excluded due to low quality of staining. A genotype-blind operator performed image acquisition and processing.

### Genome-wide SNP assay

The SNP&SEQ technology platform at Uppsala University performed the SNP genotyping. Illumina Mouse Low Density (LD) Linkage Panel, containing 377 SNPs, was selected from the Wellcome-CTC Mouse Strain SNP Genotype Set (Fig. 2b); it was used with the Golden Gate Genotyping Assay protocol^50^. To find parental-specific alleles, the genomes of C57Bl/6 and SwissOF1 founders were genotyped. For an SNP to be parental-specific, it should vary between C57Bl/6 and SwissOF1, but not within the population of outbred SwissOF1 used for intercrossing. Following these criteria, 126 of the 377 SNPs were parental-specific. Among the F2-En1+/− mice, one individual had missing genotype data at all markers and was removed from the analysis. No other individuals lacked a notable number of markers (Suppl. Fig. 2a, b) and none of the markers had missing genotypes for a notable number of individuals (Suppl. Fig. 2c). Due to the selection of F2-En1+/− individuals,markers adjacent to the En1 locus (chr. 1, 77–162 Mb) showed distorted segregation patterns, with significant deviation from the expected 1:2:1 distribution, and markers with p-value (<1E-5) were removed from the analysis (Suppl. Fig. 2d). No other markers had a significantly distorted allele segregation pattern according to chi-square test for Mendelian segregation (Fig. 1c). We thus have a good genomic coverage for linkage analysis, but the model with heterozygous transgene selection prevents detection of potential QTLs near the En1 locus.

### Single-QTL analysis

The data were analyzed using R/qtl (v1.34–17)^37^ to identify gene regions linked to neurodegeneration. *Scanone* was used for single QTL analysis. For expectation-maximization (EM) and Haley-Knott methods, the genotype probabilities were calculated with 0.5 cM distance and a genotyping error rate set at 0.001. For multiple imputation the genotype was simulated with 1000 simulation replicates, step length of 0.5 cM and error probability of 0.001. Significance thresholds for LOD scores were obtained by permutation test, with 1000 permutations using Haley-Knot-regression. Animals included in the QTL analysis for the number of nigral DNs were 96, for swellings per remaining nigral DN were 94 and for swellings average size were 104.

### Multiple-QTL analysis

The single-QTL analysis is based on the assumption that there is only one QTL, and the scan is performed at one locus at a time. To increase the power to detect QTLs with additive or epistatic effects, we performed multiple-QTL analysis. The multiple QTL models were fitted starting with the locus with the highest LOD score in the single-QTL model. The models were iteratively built by scanning for interactive and additive loci using addqtl with Haley-Knot regression. Fitqtl was used to fit the models. Loci and interactions with a p-value less than 0.05 in the drop-one-term ANOVA were kept in the model and used in scanning for additional loci. Genotype probabilities were calculated with a step length of 0.1 cM and error probability of 0.001. To estimate the positions of QTLs in the model, we calculated the approximate 95% Bayes credible intervals^53^. Significance thresholds for the multiple QTL models were estimated by permuting randomly selected positions 5000 times and taking the 95th percentile LOD score. This was done for the full model LOD scores as well as for individual QTL LOD scores in the model. The full model significance threshold thus is a measure of significance of the full models, while the QTL LOD score significance threshold gives an estimation of the contribution of a specific QTL to the respective model.

### Statistical Analyses

Statistical tests for the quantification of TH-positive DNs in the SNpc and axonal swellings in the striatum and correlations between those were performed using GraphPad Prism software (version 6, GraphPad, La Jolla, CA). Differences between all seven groups used for stereological estimation and the three groups used for axonal swellings quantification were analyzed using a one-way ANOVA with Tukeys’s multiple comparisons test; statistical significance was set at *p-*value < 0.05 and values are expressed as mean ± standard deviation (SD). Correlation analyses were performed using the Pearson correlation coefficient (r), statistical significance was set at *p-*value < 0.05, and a 95% confidence interval was used.

## Additional Information

**How to cite this article**: Kurowska, Z. *et al*. Identification of Multiple QTLs Linked to Neuropathology in the Engrailed-1 Heterozygous Mouse Model of Parkinson’s Disease. *Sci. Rep.* **6**, 31701; doi: 10.1038/srep31701 (2016).

## Supplementary Material

Supplementary Information

## Figures and Tables

**Table 1:** LOD-score, position and interactions of QTLs in the three respective models.

Phenotype	QTL	LOD score	Chr	Bayesian credible interval (cM)	Nearest marker	Interactions	Full model statistics	Variance explained
Number of DNs in SNpc	En1a	13.1	16	28.9–30.4	rs4180773	En1c	p = 2.4E-09 LOD = 28.3 (12.2)	74%
	En1b	11.3	14	27.2–34.9	rs3695574	En1d		
	En1c	10.5	14	14.3–22.4	rs3695383	En1a. En1g		
	En1d	10.0	2	28.2–29.0	rs13476490	En1b		
	En1e	8.8	2	36.0–67.8	rs3658729	En1f		
	En1f	7.7	11	69.5–72.9	rs13481230	En1e		
	En1g*	5.8	9	5.9–12.0	rs13480107	En1c		
	En1h*	3.0	18	31.7–46.5	rs6320743			
Number of axonal swellings per DN	En1i	25.2	15	19.9–22.3	rs3674266	En1k, En1j, En1l	p = 1.7E-12 LOD = 32. (10.1) 4	80%
	En1j	20.7	4	10.8–16.2	rs3653593	En1i, En1m		
	En1k	15.1	6	58.1–68.4	rs3152403	En1i		
	En1l	13.2	9	61.2–63.4	rs3694903	En1i		
	En1m	12.7	3	76.8–83.1	rs3724562	En1j		
	En1n*	4.3	18	20.1–36.0	rs3669543			
	En1o*	4.1	2	68.3–83.8	rs6376291			
Average size of axonal swellings	En1p	21.0	6	72.1–77.6	rs6387265	En1q, En1r, En1s	p = 7.0E-11 LOD = 30.2 (14.1)	74%
	En1q	12.1	15	23.9–30.9	rs3699312	En1p		
	En1r	10.8	14	69.2–78.4	rs3698545	En1p		
	En1s	10.3	2	34.7–42.7	rs13476507	En1p		
	En1t*	8.1	13	61.2–69.7	rs6316705	En1u		
	En1u*	7.8	7	15.7–36.2	rs3696018	En1t		
	En1v*	3.8	4	9.8–22.8	rs3653593			
	En1x*	3.7	14	14.2–29.2	rs3695383			

Full model statistics include p-value and LOD scores for the full QTL model with significance LOD threshold in parentheses.

^*^Individual QTL below the respective genome-wide significance LOD threshold (Number of DNs in SNpc = 7.1, Number of axonal swellings per DN = 4.8, Average size of axonal swellings = 8.7). LOD: Logarithm of odds, QTL: quantitative trait locus, DN: Dopaminergic neuron, SNpc: Substantia nigra pars compacta.
