# Supplementary material for: Identification of Multiple QTLs Linked to Neuropathology in the Engrailed-1 Heterozygous Mouse Model of Parkinson’s Disease
Source: Sci Rep. 2016 Aug 23;6:31701. doi: 10.1038/srep31701 (PMC4994027; doi:10.1038/srep31701)
Supplement: Supplementary Information [file srep31701-s1.pdf]

Identification of Multiple QTLs Linked to Neuropathology in the Engrailed1 Heterozygous Mouse Model of Parkinson's Disease

Zuzanna Kurowska[a, b, c, #], Michael Jewett[a, #], Per Ludvik Brattås[a, #], Itzia Jimenez-Ferrer[a], Xuyian Kenéz[a], Tomas Björklund[d], Ulrika Nordström[e], Patrik Brundin[f] and Maria Swanberg[a, *]

a. Neurodegeneration and Inflammation Genetics Unit, Wallenberg Neuroscience Center, Department of Experimental Medical Science, Lund University, BMC A10, Sölvegatan 17, 221 84 Lund, Sweden
b. Department of Neurosciences, Lerner Research Institute, Cleveland Clinic, 9500 Euclid Avenue, Cleveland, Ohio 44195, USA
c. Renovo Neural Inc., 10000 Cedar Avenue, Cleveland 44106, OH, USA
d. Molecular Neuromodulation Unit, Lund University, Biomedical Center A10, Sölvegatan 17, 221 84 Lund, Sweden
e. Department of pharmacology and clinical neuroscience, Byggnad 6M NUS, Umeå University, 901 87 Umeå, Sweden
f. Laboratory of Translational Parkinson's Disease Research, Center for Neurodegenerative Science, Van Andel Research Institute, 333 Bostwick Ave, N.E., Grand Rapids, MI 49503, USA

Supplementary Information

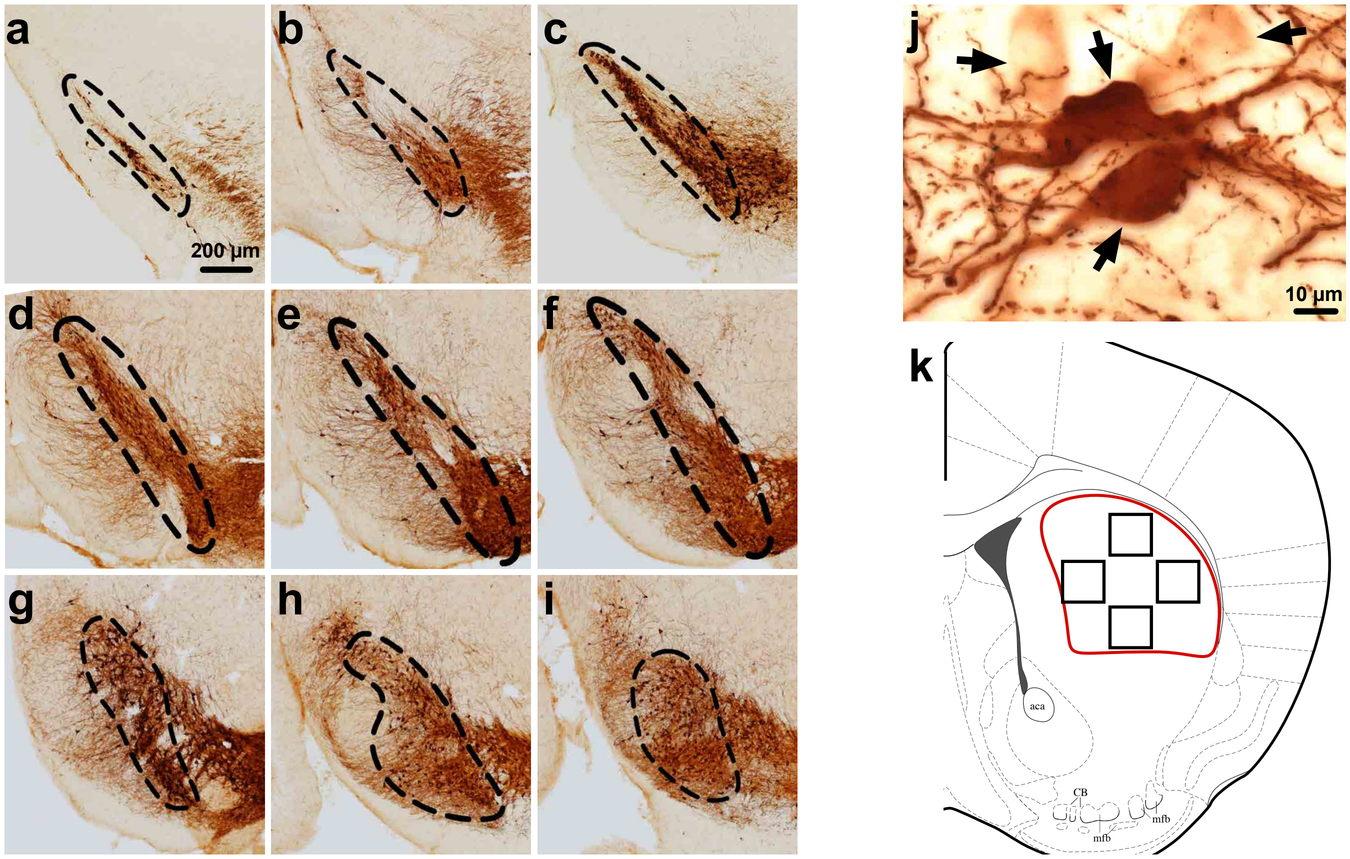

Supplementary Figure S1. Representative sampling areas for the analyses of TH-stained DNs and axonal swellings. A-I. Representation of every 3rd coronal section, spanning the SNpc region (-3.78 to -2.7 mm from bregma), organized rostro-caudally. SNpc region used for stereological estimation of total number of TH-positive DNs is marked in red. J. Examples of nigral TH-positive neurons included (black arrows) in the counting. K. Representation of the sampling scheme for the quantification of the axonal swellings in the dorso-lateral caudate putamen of striatum. Red line shows the dorso-lateral part where the 4 images (black rectangles) were taken.

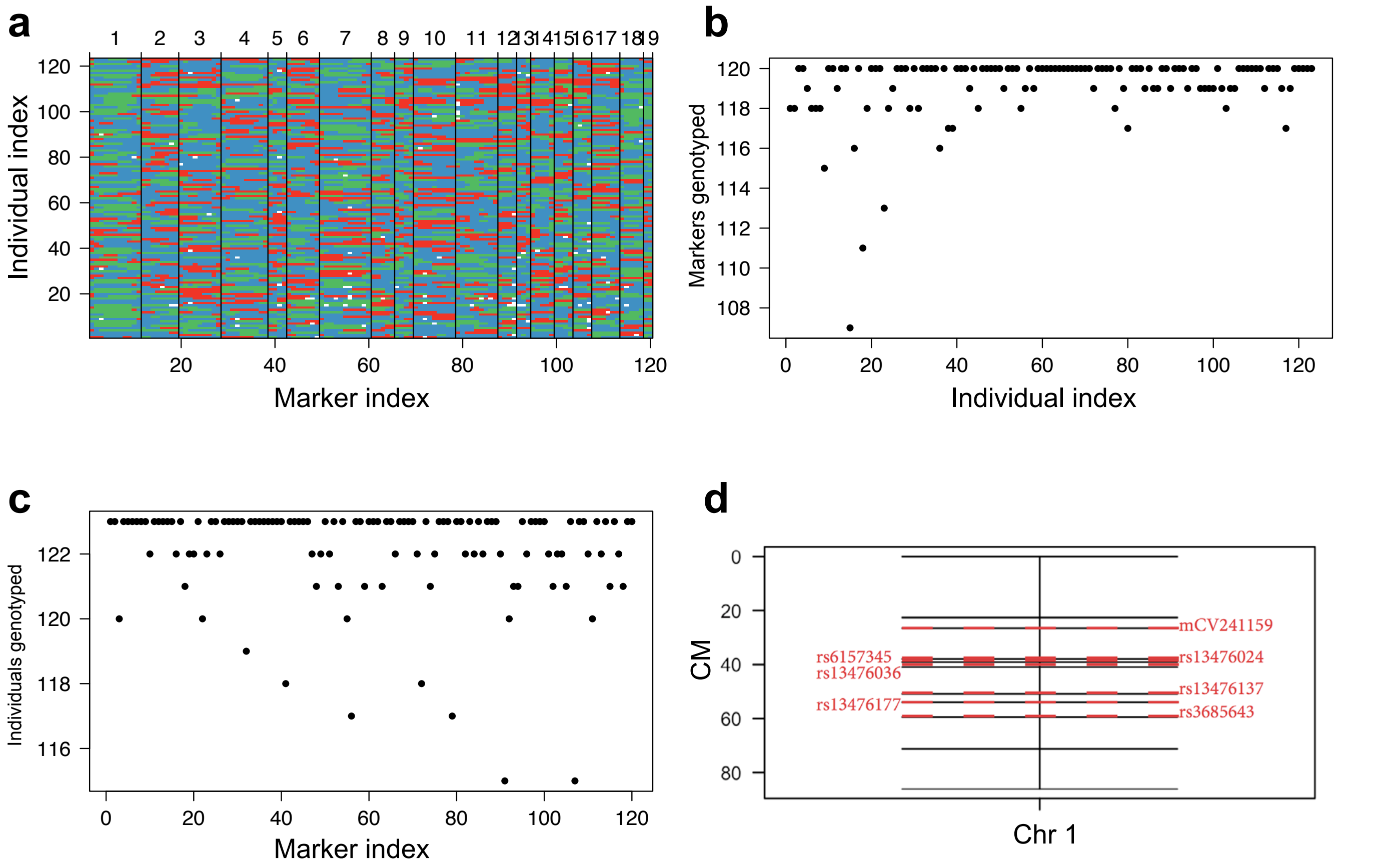

Supplementary Figure S2. Genomic coverage. A. Genotype data for each individual and each marker in the SNP genotyping. Red: Bl/Bl; Blue: Bl6/Sw; Green: Sw/Sw; B. Number of markers genotype for each genotyped individual; C. Number of individuals genotyped at each marker; D. Genetic map of chromosome 1. Red markers were removed from the analysis due to significantly distorted segregation patterns.

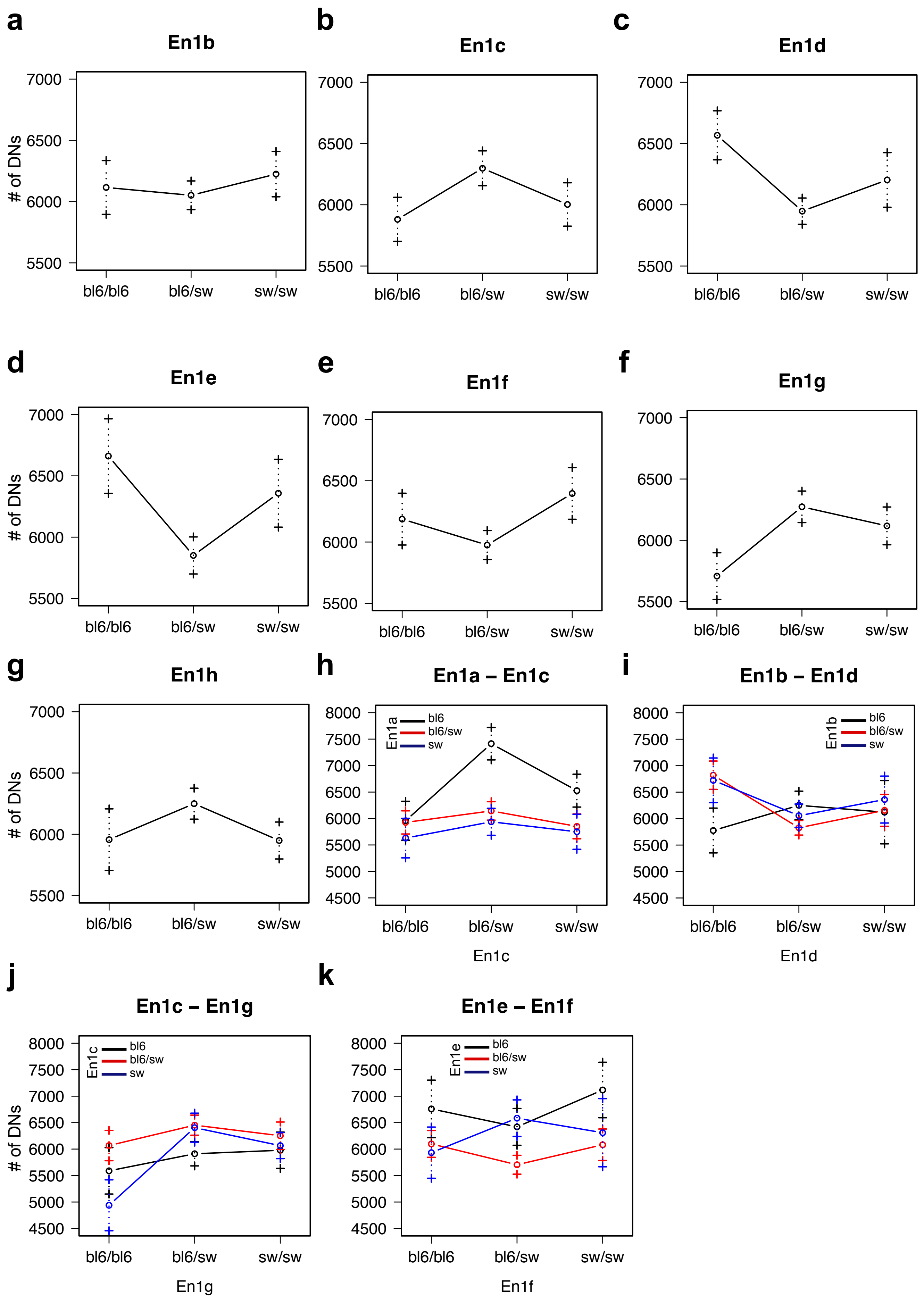

Supplementary Figure S3. QTLs linked to number of DNs. A-G. Effectplots for En1b – En1h; H-K. Interaction plots for the significant interactions for DN counts.

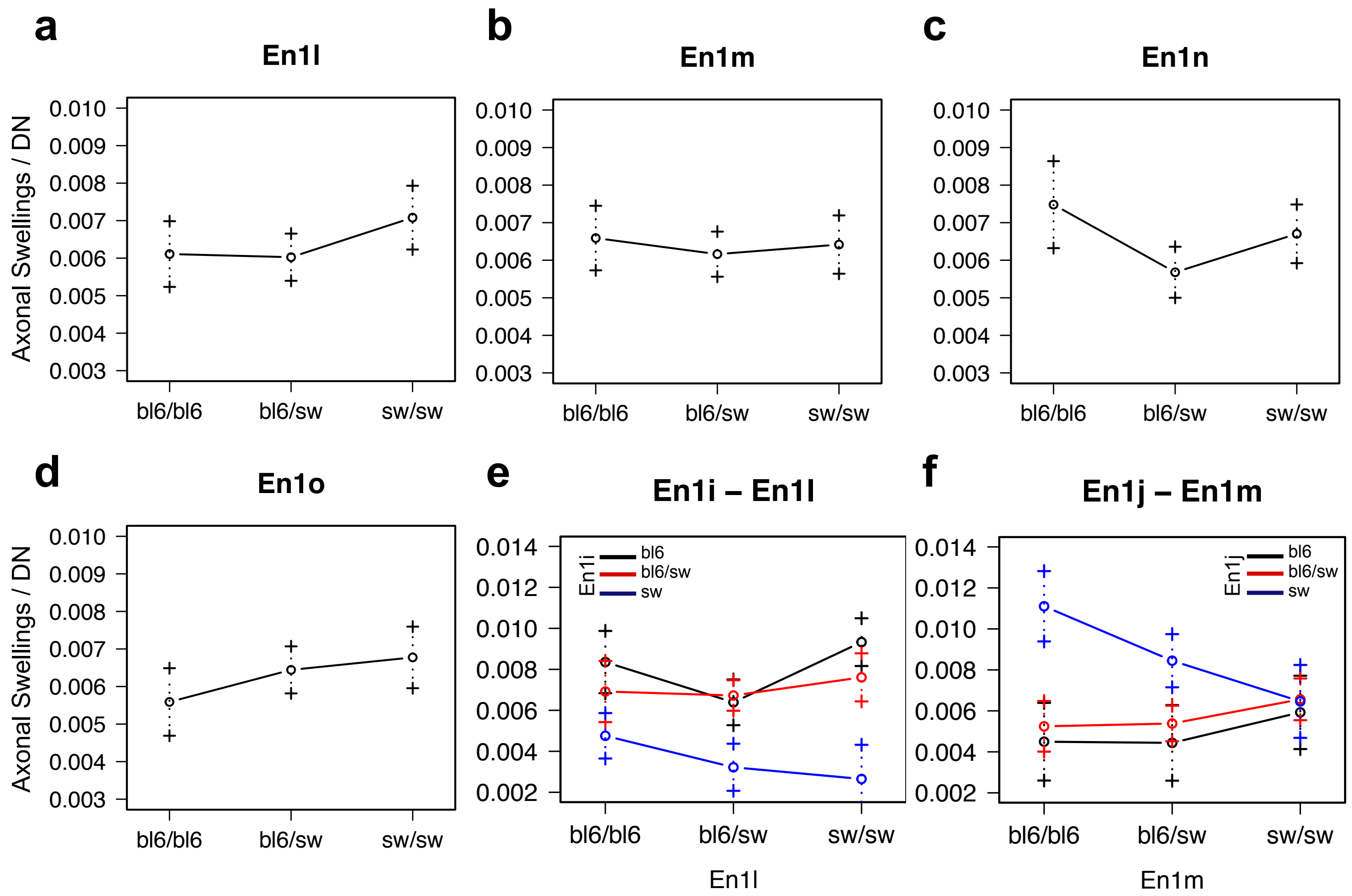

Supplementary Figure S4. QTLs linked number of axonal swellings per number of remaing DNs. A-D. Effectplots for En1l, m, n and o. E-F. Interaction plots for significant interactions between En1i-En1l and En1j-En1m.

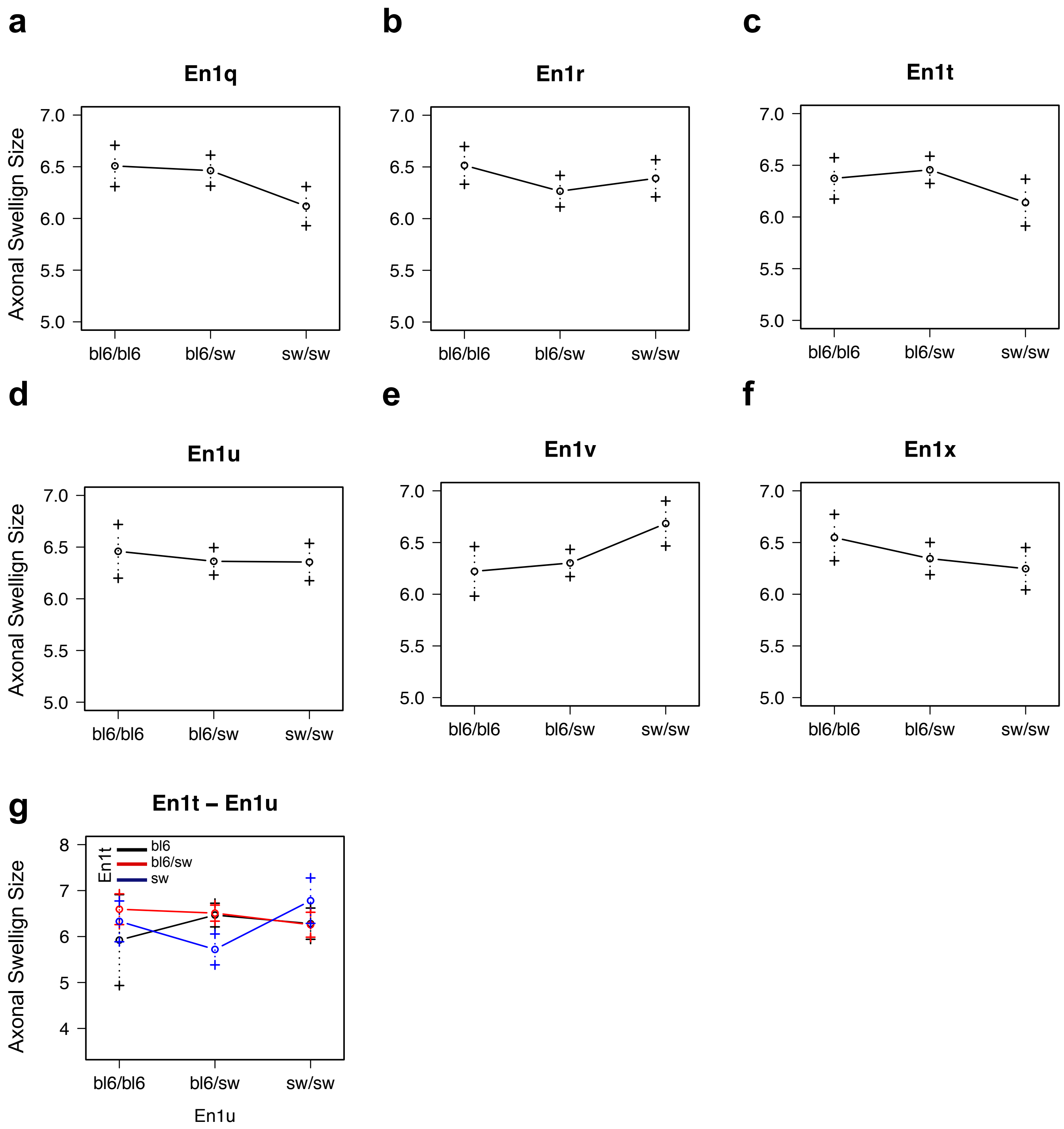

Supplementary Figure S5. QTLs linked to size of axonal swellings. A-F. Effectplots for En1q, r, t, u, v and x; G. Interaction plot for En1t-En1u.
